# Formate-Dependent Microbial Conversion of CO_2_ and the Dominant Pathways of Methanogenesis in Production Water of High-temperature Oil Reservoirs Amended with Bicarbonate

**DOI:** 10.3389/fmicb.2016.00365

**Published:** 2016-03-22

**Authors:** Guang-Chao Yang, Lei Zhou, Serge M. Mbadinga, Jin-Feng Liu, Shi-Zhong Yang, Ji-Dong Gu, Bo-Zhong Mu

**Affiliations:** ^1^State Key Laboratory of Bioreactor Engineering and Institute of Applied Chemistry, East China University of Science and TechnologyShanghai, China; ^2^Shanghai Collaborative Innovation Center for Biomanufacturing TechnologyShanghai, China; ^3^School of Biological Sciences, The University of Hong KongHong Kong, China

**Keywords:** bicarbonate, oil reservoirs, stable isotope technique, CDCS, methanogenesis, CO_2_ conversion

## Abstract

CO_2_ sequestration in deep-subsurface formations including oil reservoirs is a potential measure to reduce the CO_2_ concentration in the atmosphere. However, the fate of the CO_2_ and the ecological influences in carbon dioxide capture and storage (CDCS) facilities is not understood clearly. In the current study, the fate of CO_2_ (in bicarbonate form; 0∼90 mM) with 10 mM of formate as electron donor and carbon source was investigated with high-temperature production water from oilfield in China. The isotope data showed that bicarbonate could be reduced to methane by methanogens and major pathway of methanogenesis could be syntrophic formate oxidation coupled with CO_2_ reduction and formate methanogenesis under the anaerobic conditions. The bicarbonate addition induced the shift of microbial community. Addition of bicarbonate and formate was associated with a decrease of *Methanosarcinales*, but promotion of *Methanobacteriales* in all treatments. *Thermodesulfovibrio* was the major group in all the samples and *Thermacetogenium* dominated in the high bicarbonate treatments. The results indicated that CO_2_ from CDCS could be transformed to methane and the possibility of microbial CO_2_ conversion for enhanced microbial energy recovery in oil reservoirs.

## Introduction

In recent years, increasing atmospheric CO_2_ and the resulting climate problem become the focus of global issues. A total of 3.2 gigatonnes of CO_2_ is released by the combustion of fossil fuels each year and has led to an increase of atmospheric CO_2_ concentrations from 280 ppm in 18th century to 383 ppm in Glueck et al. (2010). Three mainly strategies to reduce CO_2_ emission and building up are: reducing the CO_2_ production, expanding the CO_2_ utilization, and CO_2_ sequestration and storage (Schrag, 2007; Yang et al., 2008). The major approaches to decrease the CO_2_ production include improvement of energy efficiency or employment of cleaner technologies. Based on energy utilization and economic input, CO_2_ is chemically relatively stable and a non-attractive raw material. Carbon dioxide capture and storage (CDCS) is regarded as a potential and practical method to reduce the CO_2_ emission into atmosphere. The injection of CO_2_ into the oil reservoirs and geosystems may not only enhance the oil recovery (EOR) but also store about two-thirds of the CO_2_ in underground systems (Mikkelsen et al., 2010; Dressel et al., 2011; Momeni et al., 2012). Injected CO_2_ could be an important factor and affects the microbial metabolism and ecophysiology in storage environment (Delgado et al., 2012; Lin et al., 2013). Injected CO_2_ could alter the microbial community and the methanogenic pathways (Mayumi et al., 2013) and raise the bicarbonate concentration in oil reservoirs (Kirk, 2011).

The injection of CO_2_ from CDCS can be transformed into methane by microorganisms for energy recovery as value-added options, but electron donors are essential for the process (Hattori et al., 2001; De Bok et al., 2004). Formate and H_2_ are known as carriers for interspecies electron transfer and CO_2_ can be reduced with formate and H_2_ as electron donors through CO_2_-reducing microorganisms in anoxic environments (Dolfing et al., 2008). Interconversion of H_2_ and CO_2_ to formate by the microorganism at ambient conditions has been reported (Schuchmann and Müller, 2013). The *Thermococcus* sp. are capable of formate oxidation with H_2_ production alone at high-temperature (Kim et al., 2010). Many species of microorganisms can grow on formate as a sole methanogenic substrate (Wood et al., 2003; Oren, 2014). Formate can be used by some types of methanogens with formate dehydrogenase alone for H_2_ production and methane production (Lupa et al., 2008). Formate plays an important role as H_2_ storage compound for CO_2_ reduction in subsurface formation.

Formate is an important metabolite in anaerobic alkane oxidation in *Desulfatibacillum alkenivorans* AK-01 (Callaghan et al., 2012). Formate also plays an important role as intermediates in syntrophic butyrate or propionate oxidization (Hattori et al., 2001). Hydrogen production based on formate oxidation has been described before (Bagramyan and Trchounian, 2003; Meshulam-Simon et al., 2007). And both thermophilic and mesophilic communities with a formate-oxidizing bacterium and a hydrogenotrophic methanogen have been constructed for demonstration of syntrophic growth on formate (Dolfing et al., 2008). Formate serve as important degradation intermediate of alkanes and precursor for methanogenesis in oil reservoirs, but very little is known about formate metabolism before.

The whole oil reservoir can be regarded as an anaerobic bioreactor with variety of specialized microorganisms and has the potential for bioconversion of CO_2_ (Liu et al., 2015). However, very little is known about the fate of sequestrated CO_2_ and the anaerobic metabolic pathway of formate in oil reservoir. In this study, the treatments were constructed with production water of high-temperature oil reservoirs amended with formate and different concentrations of C-13 labeled bicarbonate. Microbial community was analyzed based on 16S rRNA gene after incubation and high levels of methane generated. In addition, stable isotope technique was introduced to detect the fate of injected CO_2_. The objectives of this study were to provide evidence on the possibility of microbial conversion of CO_2_ and metabolic pathways of formate.

## Materials and Methods

### Preparation of Inoculum and Enrichment Cultures

The inoculum for the culture experiments was collected from water-flooded oilfield production water of Ba 18 block of Baolige, Huabei Oilfield in China, and cultured under anaerobic conditions at 55°C in the dark. Physicochemical characteristics of the inoculum is shown in **Supplementary Table S1**. About 2 mL of inoculum was transferred aseptically into a serum bottle (120 ml internal volume) with 50 ml of basal medium containing (g/L): NaCl, 0.20; MgCl_2_.6H_2_O, 1.20; CaCl_2_.2H_2_O, 0.10; NH_4_Cl, 0.25; KH_2_PO_4_, 0.75; K_2_HPO_4_, 1.16; KCl, 1.30; rezasurin, 0.0001. Vitamin stock solution and trace element stock solution of 1.0 (mL/L) and Na_2_S.9H_2_O (0.50 g/L) were added into the medium and the final pH of the basal medium was adjusted to 7.2. The detailed composition of the vitamin stock solution and trace element stock solution was described before (Wang et al., 2012b).

^13^C-bicarbonate was introduced with a final concentration of 0, 30, 60, and 90 mM. Formate was added as the carbon source and electron donor at 10 mM. Basal medium with different concentrations of bicarbonate without added formate was used as blank. Treatments were abbreviated accordingly as S0, S30, S60, and S90 for different bicarbonate concentrations. All sets of experiments were conducted in triplicate. During the operation, the serum bottles were sealed with pure N_2_ gas and removed the O_2_ from the systems. All the microcosms were incubated at 55°C in the dark.

### Chemical Analysis

Gas composition (CH_4_, CO_2_, and H_2_) of headspace was measured using a Gas Chromatograph (GC112A, Shanghai Precision and Scientific Instrument, CO., Ltd, China) with a thermal conductivity detector (TCD) and a flame ionization detector (FID). The detailed method was followed as described before (Mbadinga et al., 2012). Formate and acetate were detected and quantified by Ion Chromatograph (IC DX-600, Dionex, CO., USA) with IonPac AS11-HC analytical column (4 mm × 250 mm) and ASRS 300 suppressor. The mobile phase was 2 mM of NaOH.

### DNA and 16S rRNA Gene Amplification

Five mL of the culture samples were taken and concentrated by centrifugation at 12000 × *g* for 20 min at 4°C after 180 days cultivation. Total community DNA was extracted from the microbial biomass using the genomic DNA Kit (AxygenBiosciences, Inc., USA). Partial 16S rRNA genes of bacteria and archaea were amplified as previously described with primer 8F/805R (Zhou et al., 2013) and 340F/1000R (Gantner et al., 2011), respectively. Polymerase chain reaction (PCR) was performed in a 25 μl reaction volume containing 12.5 μM of each primer (1 μl), 50 ng of template DNA (2 μl), 12.5 μl of 2 × PCR master mix (Lifefeng Biotechnology, Shanghai, China) and 8.5 μl ddH_2_O. PCR programs were as follows: an initial denaturation step at 95°C for 5 min, followed by 32 cycles of 94°C for 45 s, 59°C for 40 s, and 72°C for 50 s, with a final elongation step at 72°C for 10 min. Genes were amplified on a Peltier Thermal Cycler (Bio-Rad, CO., USA).

### Construction of 16S rRNA Genes Libraries and Phylogenetic Analysis

Polymerase chain reaction products were cloned with a pMD 19-T simple vector kit (TaKaRa Bio, Inc., Japan) after checking by 1.8% (agarose) gel electrophoresis. Clones were randomly selected from plates and sequenced on an ABI 377 automated sequencer. The 16S rRNA sequences were checked and phylogenetic trees were generated with the protocol described before (Wang et al., 2012a). Operational taxonomic units (OTUs) were defined with the similarity of more than 97%. Phylogenetic trees of 16S rRNA genes retrieved from the original inoculum, S0, S30, S60, and S90 samples, were constructed. Sequences were performed by MEGA 5 with OTU identity of 97%. The topology of the tree was obtained with the neighbor-joining method. Bootstrap values (*n* = 1000 replicates) of ≥75% are showed.

### Data Analysis of Stable Isotope

Carbon-13 isotopic compositions of CH_4_ and CO_2_ were determined with isotope ratio mass spectrometer (IRMS Delta V PLUS, Thermo Scientific, CO., USA). The samples were concentrated with PreCon connector to reduce dose. The standard international delta notation for reporting isotopic value ratios were relative to the VPDB standard. The references gas was CO_2_ and δ^13^ of C_PDB_ was –23.73‰, which was calibrated by stable isotope of carbon in charcoal black. The system was performed with ISODATNT software.

### Thermodynamic Calculations

Gibbs free energy data for thermodynamic calculations with different temperatures (**Table 1**) were taken from (Amend and Shock, 2001). Δ*G*°_T_ is standard Gibbs free energy at temperature T and Δ*G*°′_T_ is modified with the protons from Δ*G*°_T_. The change of Gibbs free energy (Δ*G*′_T_) for the reaction is calculated with the formula: Δ*G*′_55_ = Δ*G*°′_55_ – RTln([R]^a^/[P]^b^), R and P are the abbreviations of reactants and products, respectively; a and b are the stoichiometric numbers of each composition.

**Table 1:** Gibbs free-energy changes for the feasible reactions of formate and hydrogen.

Reaction	Δ*G*° 25°C (kJ/mol)	Δ*G*° 55°C (kJ/mol)	Δ*G*°′55°C (kJ/mol)
CO_2_ reduction
HCO_3_^-^+4H_2_+H^+^ → CH_4_+3H_2_O	-175.32	-168.56	-124.58
Acetate formation
4HCOO^-^ +H^+^ → CH_3_COO^-^+2HCO_3_^-^	-139.69	-137.64	-93.66
Formate methanogenesis
4HCOO^-^+H_2_O+H^+^ → CH_4_+3HCO_3_^-^	-170.84	-172.51	-128.53
Syntrophic formate oxidation
HCOO^-^+H_2_O → HCO_3_^-^+H_2_	1.12	-0.92	-0.92


*Formula: Δ*G*°′ = Δ*G*°+m2.303RTlog10^-7^(m is the net number of protons formed in the equation) Δ*G*°T: standard Gibbs free energy at temperature T.*

### Nucleotide Sequence Accession Numbers

The partial gene sequences for bacteria and archaea obtained from the clone libraries were submitted to GenBank database under accession numbers KR049100–KR049155 and KR017718–KR017745, respectively.

## Results

### Methane and Hydrogen Production

**FIGURE 1:**
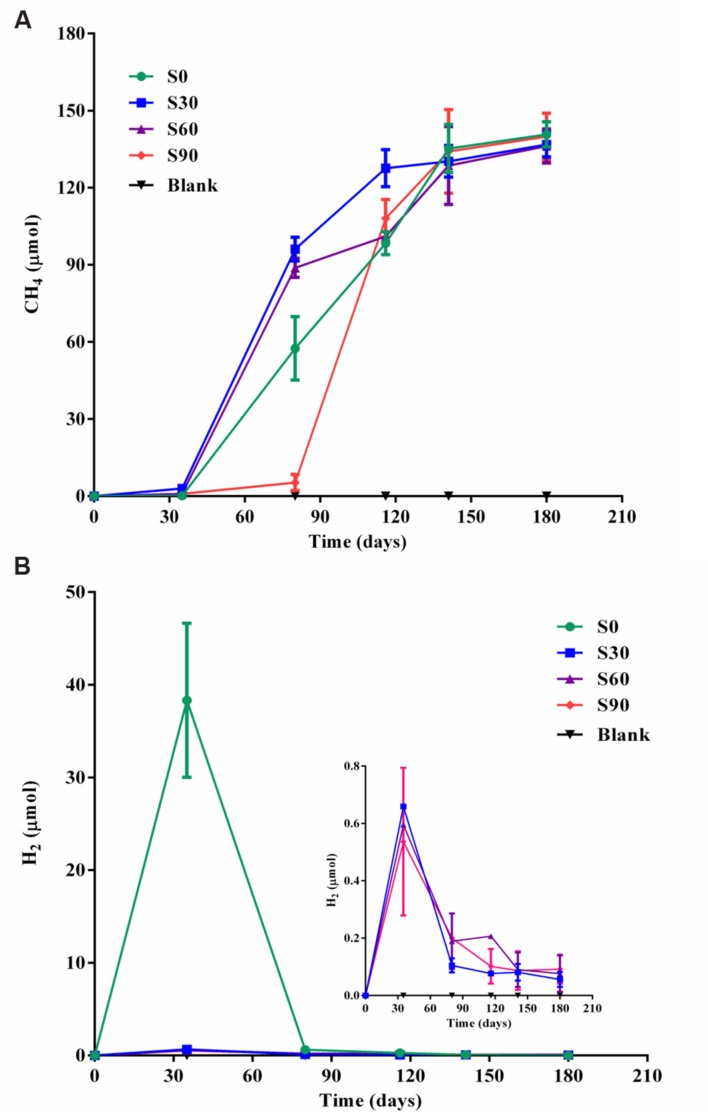
**Methane and hydrogen production in the cultures.** Methane production **(A)** and hydrogen production **(B)** in serum bottle maintained under anaerobic conditions. Blank are the cultures with different concentrations of bicarbonate without added formate. S0, S30, S60, and S90 treatments were the cultures with formate and different concentrations of bicarbonate (0, 30, 60, and 90 mM, respectively).

Methane production increased after the initial 35 days of incubation under strictly anaerobic conditions, and an obvious lag period was observed in S90 compared with the other treatments (**Figures 1A,B**). The rate of methane production in S90 was about 1.95 pmol d^-1^ ml^-1^ during the culture of 35∼80 days, while it was about 25.5, 41.4, and 39.1 pmol d^-1^ ml^-1^ in S0, S30, and S60, respectively. After 180 days of incubation, methane production in all the treatments reached approximately 136 μmol/bottle, corresponding to almost exhaustion of the 500 μmol/bottle of formate introduced into the medium (**Supplementary Table S2**). Methane production was nearly equal to the formate consumed indicating that almost all of the methane produced was by formate reduction directly or indirectly. The ultimate methane production rate of all the samples was about 19.6 pmol d^-1^ ml^-1^. An obvious hydrogen production was found in S0 samples and subsequently depleted, and little hydrogen was detected in other samples amended with bicarbonate addition after 35 days and decreased with the further incubation of the cultures (**Figure 1B**), which showed the same trend with the S0 sample. The addition of bicarbonate decreased the amount of hydrogen production. There was no methane or hydrogen detected in all the blank treatments without added formate.

### Dynamics of Microbial Community

**FIGURE 2:**
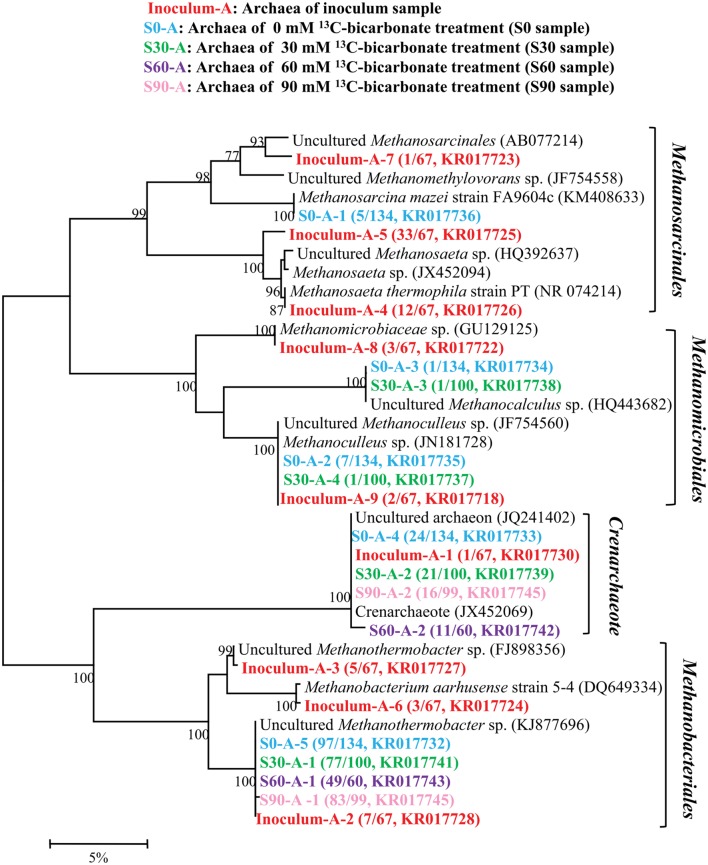
**Phylogenetic tree of archaeal genes retrieved from five samples (shown with different color).** Sequences were performed by MEGA 5 with OTU identity of 97%. The topology of the tree was obtained with the neighbor-joining method. Bootstrap values (*n* = 1000 replicates) of ≥75% are showed. Numbers in brackets are the appearance frequencies of the identical sequences in clones and the accession number.

16S rRNA clone libraries were constructed to analyze the microbial community under different bicarbonate concentrations compared with the original inoculum. A total of 460 sequences were obtained and then used to analyze the archaeal composition in inoculum, S0, S30, S60, and S90 samples and 9, 5, 4, 2, and 2 OTUs were resulted with 97% similarity, respectively (**Figure 2**). All the archaeal sequences belong to *Methanomicrobiales*, *Methanobacteriales*, *Methanosarcinales*, and *Crenachaeota*. Those affiliating with *Methanomicrobiales* and *Methanobacteriales* are CO_2_-reducing methanogens while *Methanosarcinales* have a variety of methanogenic biochemical pathways. *Crenachaeota* is known as ammonia-oxidizing bacteria (Hatzenpichler et al., 2008) and have no reported information about methanogenesis. The original inoculum contained the most varieties of archaea among all the five samples. With the addition of formate and increase in bicarbonate concentrations, the archaeal varieties decreased and only *Methanobacteriales* and *Crenachaeota* were detected in S60 and S90. In the original inoculum, *Methanosarcinales* were dominated (68.7%) with 3 OTUs (Inoculum-A-4, Inoculum-A-5, and Inoculum-A-7) and 3 OTUs (Inoculum-A-2, Inoculum-A-3 and Inoculum-A-6) with close identities to *Methanobacteriales* (22.4%), 2 OTUs (Inoculum-A-8 and Inoculum-A-9) belonged to *Methanomicrobiales* (7.5%) were also detected and OTU Inoculum-A-1 (1.4%) clustered into *Crenachaeota*. *Methanobacteriales* (72.4%∼83.8%) were dominated in all the culture treatments amended with formate. *Methanomicrobiales*, *Methanobacteriales*, *Methanosarcinales* and *Crenachaeota* were all detected in the S0 and S30 samples, but only *Methanobacteriales* and *Crenachaeota* were detected in the treatments (S60 and S90) with high bicarbonate concentrations.

**FIGURE 3:**
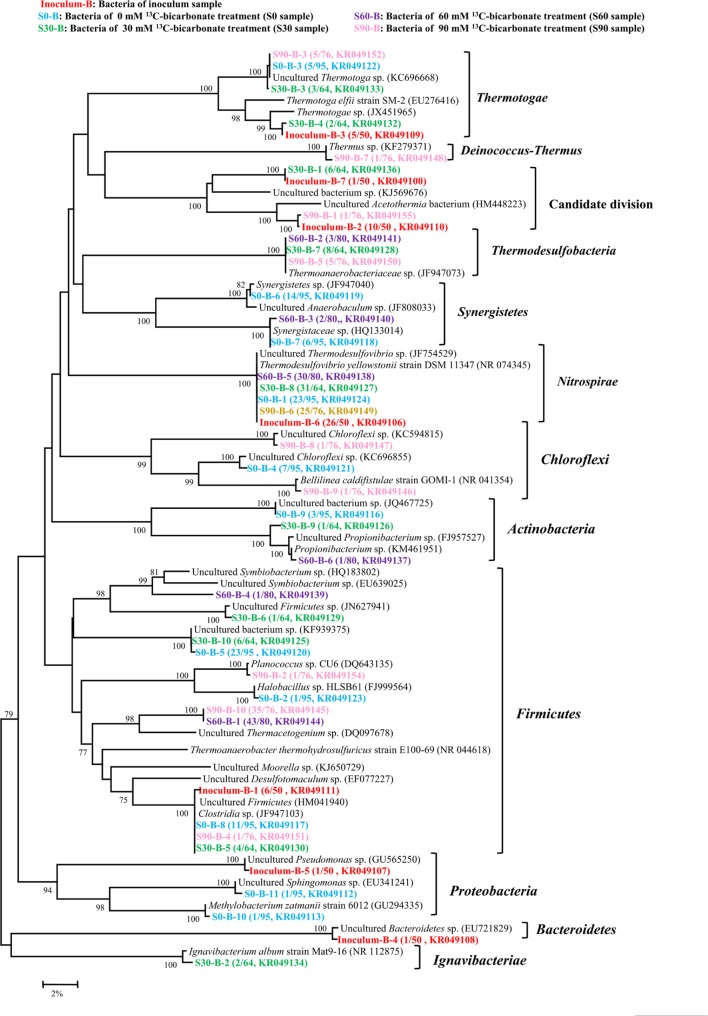
**Phylogenetic tree of bacterial genes retrieved from five samples (shown with different color).** Sequences were performed by MEGA 5 with OTU identity of 97%. The topology of the tree was obtained with the neighbor-joining method. Bootstrap values (*n* = 1000 replicates) of ≥75% are showed. Numbers in brackets are the appearance frequencies of the identical sequences in clones and the accession number.

By using bacterial gene specific primers, a total of 365 sequences were obtained and then analyzed with the 97% similarity to construct a phylogenetic tree about bacteria, and 7, 11, 10, 6, and 10 OTUs were found in the inoculum, S0, S30, S60, and S90 samples, respectively (**Figure 3**). Bacteria showed high varieties in all the five samples and most sequences belonged to *Thermotogae*, *Synergistetes*, *Acetothermia*, *Nitrospirales*, and *Firmicutes*. *Thermodesulfovibrio* belonged to *Nitrospirales* and *Clostridia* were found in all the five samples. *Thermodesulfovibrio* was dominated in the treatments (inoculum, S0, and S30) with low concentrations of bicarbonate. *Thermacetogenium*, a member of *Clostridia*, increased its presence in the treatments (S60 and S90) with high bicarbonate concentrations. *Thermoanaerobacteriaceae* was only detected in the treatments amended with bicarbonate addition (S30, S60, and S90).

### Stable Carbon Isotope Analysis

**FIGURE 4:**
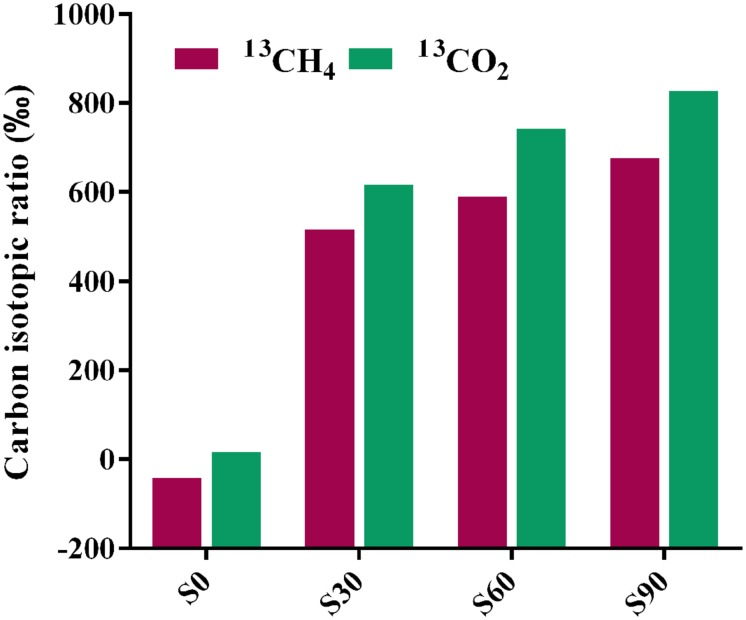
**Carbon isotopic ratios of methane and CO_2_ after incubation.** The relevant data are shown in **Supplementary Table S3**. S0, S30, S60, and S90 treatments were the cultures with formate and different concentrations of bicarbonate (0, 30, 60, and 90 mM, respectively).

Bicarbonate was added to mimic the highly buffered condition due to the injected CO_2_ and stable isotope C-13 was used to identify the fate of CO_2_ (bicarbonate form) in incubation with deep-subsurface fluid. The carbon isotopic compositions of methane and CO_2_ were detected after incubation for 180 days. In the treatment without ^13^C-bicarbonate addition, there was no obvious ^13^CH_4_ detected (**Figure 4**). But hydrogen production was detected and subsequently consumed (**Figure 1B**), indicating that H_2_ were produced from formate and then used for methane production. ^13^CH_4_ were detected in the treatments with ^13^C-bicarbonate addition (516.03∼675.46‰; **Figure 4**) and the production rate of ^13^CH_4_ increased as ^13^C-bicarbonate concentration increased, which suggested that C-13 labeled carbon dioxide (or ^13^C-bicarbonate) were reduced to ^13^CH_4_ and promoted the production rate of ^13^CH_4_. The ratio of labeled C-13 stayed as ^13^C-bicarbonate increased as injected ^13^C-bicarbonate concentration increased, while the amount of all detected C-13 and the percentage of labeled C-13 stayed as ^13^CO_2_ or ^13^CH_4_ decreased (**Table 2**). Carbon dioxide without any labeled ^13^C was detected (173.88∼383.48‰) in the culture treatments, which should be produced from formate. In the treatments amended with different concentrations of ^13^C-bicarbonate, formate was oxidized to CO_2_ and high concentration of CO_2_ with an increasing incorporation of ^13^CO_2_ and then a large fraction of ^13^CO_2_ with small fraction of CO_2_ were reduced to ^13^CH_4_ and CH_4_. (formate oxidation: HCOO^-^ + H_2_O = HCO_3_^-^ + H_2_; CO_2_ reduction: HCO_3_^-^ + 4H_2_ + H^+^ = CH_4_ + 3H_2_O). CO_2_/^13^CO_2_ also could be involved in formate methanogenesis directly by methanogens and produced CH_4_/^13^CH_4_.

**Table 2:** The fate of injected ^13^C-bicarbonate in the experiments at 180 days of incubation.

Samples	Total-^13^C (μmol)	Detected-^13^C (μmol)	^13^C-CH_4_ (μmol)	^13^CO_2_ (μmol)	^13^C-bicarbonate (μmol)
S30	1500	1383.15	68.94 (4.98%)	893.68 (64.61%)	420.53 (30.40%)
S60	3000	2390.31	78.59 (3.29%)	1253.48 (52.44%)	1058.24 (44.27%)
S90	4500	3210.23	92.84 (2.89%)	1638.02 (51.03%)	1479.37 (46.08%)


*The calculations were shown in **Supplementary Table S3**.*

### Thermodynamics of Bicarbonate-Driven Methanogenic Reactions

**FIGURE 5:**
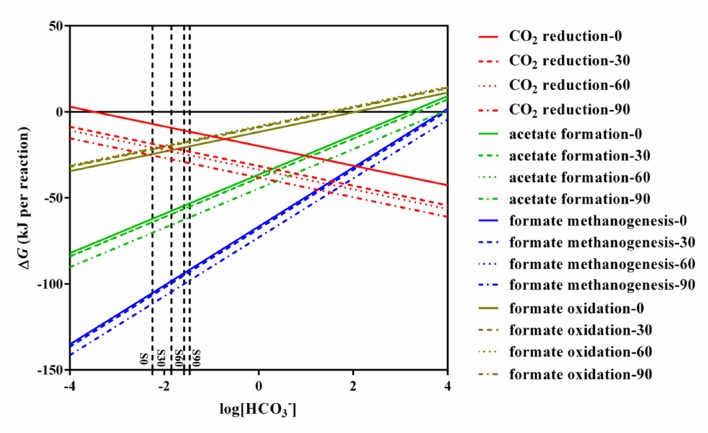
**Effects of HCO_3_^-^ on change in Gibbs free energy for CO_2_ reduction, acetate formation, formate methanogenesis, and formate oxidation.** All lines in the figure were calculated using the conditions of S0, S30, S60, S90. The detail data were shown in **Supplementary Table S2**.

Thermodynamic calculation was used to illustrate the energetically favorable metabolic pathways in the experiments with different concentrations of bicarbonate associated with microbial community. The theoretical constraints of archaea and bacteria were evaluated respectively with the data obtained in the present study with different concentration of bicarbonate. The thermodynamic calculation of different concentration of bicarbonate show the same trend. The calculations for the feasible reactions of hydrogen and formate in the sets of culture experiments amended with bicarbonate (S0, S30, S60, and S90) are as follows: CO_2_ reduction, acetate formation, formate methanogenesis, and formate oxidation (**Table 1**). The thermodynamics are shown in **Figure 5.** Acetate formation, formate methanogenesis, and formate oxidation were less energetically favorable with increasing bicarbonate concentrations and CO_2_ reduction became more energetically favorable. As other factors are fixed, CO_2_ reduction and formate oxidation are less sensitive to the change of bicarbonate concentration than formate methanogenesis and acetate formation because one mole of bicarbonate involves in per CO_2_ reduction and formate oxidation reaction. The change of Gibbs free energy (Δ*G*′_55_) calculated for the four reactions in all the treatments showed that all the reactions are exergonic under the experimental conditions and formate methanogenesis is the most favorable one. Δ*G*° 25°C of the formate oxidation is above the zero (1.12 kJ/mol) and Δ*G*° 55°C of the formate oxidation is only -0.92 kJ/mol, which are close to thermodynamic threshold (**Figure 5** and **Table 1**). Both formate methanogenesis and formate oxidation are energetically favorable in the culture conditions.

## Discussion

Oil reservoir is an extreme environment with different conditions of temperatures, pH values, pressure, salinity, thermodynamic limits and barren habitat (Kobayashi et al., 2012; Mayer and Müller, 2014). These extreme conditions require microbes thriving in community to carry out biochemical reactions collectively. The injected CO_2_ from CDCS or MEER can alter the microbial community and the metabolic pathways in deep-subsurface environments, which may dictate the fate of CO_2_ (Mayumi et al., 2013; Ohtomo et al., 2013). The endogenous microorganisms in oil reservoirs can reduce CO_2_ with the electron derived from the electron donors, which define the oil reservoirs as a bioreactor for CO_2_ bioconversion to either fixation as acetate or as methane, a source of natural gas.

### The Fate of the Injected CO_2_

Our studies showed that about 136 μmol methane produced after 180 days of incubation in each bottle and at least 516.03% was labeled in the treatments with ^13^C-bicarbonate addition, indicating that at least half of the methane produced were through the CO_2_ reduction pathway for methanogenesis. At the same time, part of injected bicarbonate were presented as ^13^C-bicarbonate in culture medium and ^13^CO_2_ of gas phase in headspace (**Table 2**). In original oil reservoir environments, residual CO_2_ from EOR could be converted to methane via methanogenesis, whereas most CO_2_ was dissolved in the formation fluids and appeared in gas phase after more than 30 years (Shelton et al., 2014). With the simulation reactor system in laboratory, injected CO_2_ could be transformed into acetate through homoacetogenesis, but methanogenesis was not detected under the condition simulating the *in situ* pressure and temperature (Ohtomo et al., 2013). Carbon dioxide can also be converted into formate through the carbon dioxide reductase or a whole cell system (Schuchmann and Müller, 2013).

High CO_2_ pressure invoke acetoclastic methanogenesis instead of syntrophic acetate oxidation coupled with CO_2_-reducing methanogenesis, when acetate and oil were added as substrates (Mayumi et al., 2013). Our results showed that when formate was added as substrate and electron donors, CO_2_ could be converted into methane through syntrophic formate oxidation coupled with CO_2_-reducing methanogenesis and formate methanogenesis. The bio-conversion rate of CO_2_ was about 19.6 pmol d^-1^ ml^-1^ in the treatments, and compared to original oil reservoir environments, the abundant supply of electron donor is essential to maintain and accelerate the process to take place. Naturally available H_2_ or electron resources for methanogenesis can be the primary requirements to improve the conversion rate of CO_2_ (Head et al., 2003; Cheng et al., 2009). And variety of enzymes (Yeates et al., 2008; Shekh et al., 2011; Alissandratos et al., 2014) and catalysts (Hull et al., 2012; Ziebart et al., 2012; Jeletic et al., 2013) can be used to convert the CO_2_ into reduced compounds under *in situ* and *ex situ* experimental conditions.

### Methanogenic Pathway Analysis in the Experiments

**FIGURE 6:**
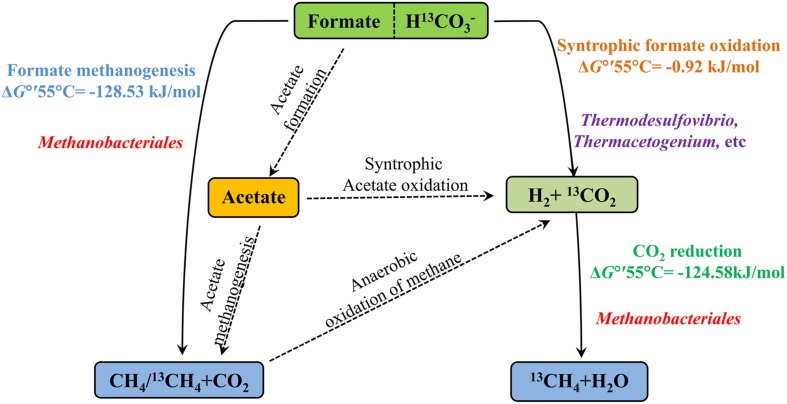
**The proposed pathways of anaerobic formate transformation and related CO_2_ conversion.** Solid lines mean the pathways were detected in the treatments and dotted lines mean not. Δ*G*°′55°C is standard Gibbs free energy at temperature 55°C and modified with protons (**Table 1**). Microorganisms are positioned according their probable role in the treatments.

Biological CO_2_ fixation is a common phenomenon in biology. Six autotrophic carbon fixation pathways have been reported (Berg et al., 2010) and most key enzymes of these six biochemical pathways have been detected in oil reservoirs (Liu et al., 2015). There are three main methanogenic pathways in oil reservoirs: CO_2_-reducing, acetoclastic and methylotrophic with different substrates: CO_2_, acetate and methyl group containing compounds, respectively (Nazaries et al., 2013) and direct interspecies electron transfer as a new model for methanogenesis has been confirmed (Rotaru et al., 2014). In this study, formate was amended into the treatments as the carbon source and electron donor. The proposed pathways of anaerobic formate transformation and related CO_2_ conversion were shown in **Figure 6.** Formate can be involved in CO_2_ conversion and methanogenesis through many kinds of pathways.

Methane production was nearly equal to the formate consumed and consistent with stoichiometric formate oxidation reaction, indicating syntrophic formate oxidation and formate methanogenesis would be involved. Hydrogen was detected in all the samples at 35 days and decreased with the further incubation of the cultures (**Figure 1B**), suggesting that hydrogen was produced and then used for the CO_2_ reduction, which also indicated that syntrophic formate oxidation coupled with CO_2_ reduction and formate methanogenesis would be responsible for methanogenesis. The hydrogen could be generated through syntrophic formate oxidation or as intermediate during the formate methanogenesis in the samples. The obvious hydrogen production was found in S0 treatments and little hydrogen was detected in other culture samples amended with bicarbonate addition, because a high concentration of hydrogen could not be accumulated in these treatments with the limitation of Gibbs free energy threshold (Δ*G*′ = 0, **Table 1**). Meanwhile, CO_2_ reduction, formate oxidation, and formate methanogenesis were also below the threshold values, indicating these reactions were energetically favorable and spontaneous in the treatments. The production and subsequent consumption of hydrogen suggest that CO_2_ reduction dominated in the systems.

Formate was once known as nutritional substrate but not for methanogenesis directly by methanogens (Tanner et al., 1989), but some genera of *Methanobacteriaceae*, which can use formate directly for methanogenesis were found (Oren, 2014). Methane with C-13 labeled was detected in the treatments with amendment of ^13^C-bicarbonate (516.03∼675.46‰), suggesting that ^13^C-bicarbonate (or ^13^CO_2_) was reduced to ^13^CH_4_ (**Figure 4**), indicating the CO_2_-reducing pathway was dominant in the experiments. Carbon dioxide without any labeled ^13^CO_2_ was detected (173.88∼383.48‰) in the treatments (**Figure 4**), which should be produced by syntrophic formate oxidation. Formate is known as a potential energy source and important degradation intermediate in ecosystem. Based on our results, syntrophic formate oxidation coupled with CO_2_ reduction and formate methanogenesis could be the possible methanogenic pathway in the experiments.

### CO_2_-Induced the Shift of Microbial Community

Oil reservoir is a typical anaerobic environment and the endogenous microorganisms in oil reservoir can degrade alkanes and produce methane (Mbadinga et al., 2011). In this study, the archaea in the inoculum had a high diversity and the ability to produce methane through CO_2_-reducing, acetoclastic and methylotrophic methanogenic pathways (**Figure 2**). However, some types of archaea were not detected in the treatments with the addition of formate and increase of bicarbonate concentrations, *Methanosarcinales* disappeared in the sample amended with 30 mM of bicarbonate, and only *Methanobacteriales* and *Crenachaeota* were detected in S60 and S90 treatments. The results indicated that *Methanomicrobiales* and *Methanosarcinales* were sensitive to the high bicarbonate concentrations. Compared to the original inoculum, *Methanobacteriales* was dominated in all the treatment cultures in the presence of formate, suggesting formate promoted the growth of the *Methanobacteriales*. *Methanobacteriales* is a kind of strict CO_2_-reducing methanogens, it also possesses formate dehydrogenase and could be candidate for formate-dependent H_2_ production and methanogenesis directly (Lupa et al., 2008), which is in agreement with the mentioned methanogenic pathway.

Bicarbonate concentration showed little impact on bacterial diversity. *Thermodesulfovibrio* belonged to *Nitrospirales* was a major group in the inoculum and all the treatments (**Figure 3**), especially in the low concentration treatments (inoculum, S0, and S30). *Thermodesulfovibrio* is a typical sulfate-reducing bacterium and grew through the reduction of sulfate with electron donors like hydrogen and formate. *Thermodesulfovibrio* and the CO_2_-reducing methanogen in co-culture experiments can produce methane syntrophically (Sekiguchi et al., 2008). In this study, *Thermodesulfovibrio* may use formate and co-work with methanogens for methane production. *Clostridia* were the most widely studied as hydrogen producer and acetogens in anaerobic environments (Drake et al., 2008; Calusinska et al., 2010), and *Thermacetogenium* belonged to *Clostridia* increased in the treatments with high bicarbonate concentrations (S60 and S90), known as a syntrophic acetate-oxidizing bacterium and acetogens (Hattori et al., 2000). However, there was no acetate accumulation (**Supplementary Table S2**) or acetoclastic methaogens (**Figure 2**) in the treatments with high bicarbonate concentrations (S60 and S90), suggesting that acetoclastic methanogenesis was not involved in the methane production of this study.

The microbial community in the experiments was capable of converting CO_2_ into methane through syntrophic formate oxidation coupled with CO_2_-reducing methanogenesis and formate methanogenesis. *Thermodesulfovibrio* and *Methanobacteriales* may play important role for the process in the system. The addition of bicarbonate can induce the shift of microbial community, especially in archaea.

In summary, under the microcosm study, most methane was produced by reducing the amended CO_2_ through the syntrophic formate oxidation coupled to CO_2_-reducing methanogenesis and formate methanogenesis with addition of bicarbonate. The results indicated that the microbial conversion of CO_2_ to methane is feasible with the microbial community in production water of high-temperature oil reservoirs. *Thermodesulfovibrio* and *Methanobacteriales* may be responsible for formate utilization and CO_2_ conversion. The results suggested that syntrophic formate oxidation coupled to CO_2_ reduction and formate methanogenesis could bealternative methanogenic pathway and gave some knowledge on formate metabolism in subsurface environment.

## Author Contributions

J-DG and B-ZM designed the experiments, G-CY did the experiments, G-CY, LZ and SM carried out the microbial analysis. J-FL and S-ZY gave the suggestion for the experiments and results analysis. G-CY prepared the manuscript with contributions from all co-authors.

## Conflict of Interest Statement

The authors declare that the research was conducted in the absence of any commercial or financial relationships that could be construed as a potential conflict of interest.
