# Supplementary material for: Formate-Dependent Microbial Conversion of CO2 and the Dominant Pathways of Methanogenesis in Production Water of High-temperature Oil Reservoirs Amended with Bicarbonate
Source: Front Microbiol. 2016 Mar 22;7:365. doi: 10.3389/fmicb.2016.00365 (PMC4801891; doi:10.3389/fmicb.2016.00365)
Supplement: Supplementary file 1 [file Table_1.DOCX]

**Supplementary Table 1.** Physicochemical characteristics of the inoculum

| Parameter | S |
| --- | --- |
| Cl^-^ (mg l^-1^) | 340.71 |
| SO_4_^2-^ (mg l^-1^) | 0.89 |
| PO_4_^3-^ (mg l^-1^) | ND |
| NO_3_^-^ (mg l^-1^) | ND |
| Na^+^ (mg l^-1^) | 1749.86 |
| K^+^ (mg l^-1^) | 7.14 |
| Ca^2+^ (mg l^-1^) | 6.07 |
| Mg^2+^ (mg l^-1^) | 1.27 |
| Mn^2+^ (mg l^-1^) | ND |
| Acetate (mg l^-1^) | 182.58 |
| Formate (mg l^-1^) | ND |

ND, not detected
