# Supplementary material for: Formate-Dependent Microbial Conversion of CO2 and the Dominant Pathways of Methanogenesis in Production Water of High-temperature Oil Reservoirs Amended with Bicarbonate
Source: Front Microbiol. 2016 Mar 22;7:365. doi: 10.3389/fmicb.2016.00365 (PMC4801891; doi:10.3389/fmicb.2016.00365)
Supplement: Supplementary file 2 [file Table_2.DOCX]

**Supplementary Table 2** The conditions at 141 days of incubation in the experiments

| Samples | Acetate  mM | Formate  mM | H_2_  mM | CH_4_  mM | CO_2_  mM | HCO_3_^-^  mM | | pH | |  |
| --- | --- | --- | --- | --- | --- | --- | --- | --- | --- | --- |
| S0 | 0.253 | 0.032 | 0.000428 | 1.694 | 2.753 | | 5.68 | | 7.2 | |
| S30 | 0.186 | 0.036 | 0.00136 | 1.593 | 20.775 | | 14.27 | | 7.1 | |
| S60 | 0.122 | 0.032 | 0.00171 | 1.686 | 21.332 | | 26.25 | | 7.1 | |
| S90 | 0.128 | 0.061 | 0.00271 | 1.778 | 28.381 | | 34.60 | | 7.2 | |
