# Supplementary material for: Formate-Dependent Microbial Conversion of CO2 and the Dominant Pathways of Methanogenesis in Production Water of High-temperature Oil Reservoirs Amended with Bicarbonate
Source: Front Microbiol. 2016 Mar 22;7:365. doi: 10.3389/fmicb.2016.00365 (PMC4801891; doi:10.3389/fmicb.2016.00365)
Supplement: Supplementary file 3 [file Table_3.DOCX]

**Supplementary Table 3** The fate of injected ^13^C-bicarbonate in the expermients at 180 days of incubation

| Samples | Total-CH_4_  (μmol) | ^13^CH_4_-Isotopic value (‰) | ^13^CH_4_  (μmol) | Total-CO_2_  (μmol) | ^13^CO_2_-Isotopic value (‰) | ^13^CO_2_  (μmol) | ^13^C-bicarbonate |
| --- | --- | --- | --- | --- | --- | --- | --- |
| S0 | 140.77 | -41.81 | ND | 192.74 | 16.53 | 3.19 | ND |
| S30 | 136.81 | 516.03 | 70.60 | 1454.73 | 616.52 | 896.87 | 420.53 |
| S60 | 136.24 | 589.06 | 80.25 | 1693.26 | 742.16 | 1256.67 | 1058.24 |
| S90 | 139.91 | 675.46 | 94.50 | 1986.65 | 826.12 | 1641.21 | 1479.37 |

ND, not detected

The ^13^C from addtion of ^13^C-bicarbonate were calculated as follows: the ^13^C values in bicarbonate addition experiments (S30, S60, S90) minus the values in experiments without bicarbonate addition (S0)
